# Practical Points

**Published:** 1899-07-08

**Authors:** 


### PRACTICAL POINTS.

### COTTAGE HOSPITALS AND SICK PAY.

*"The Chairman of a Cottage Hospital" asks: Should sick pay from a patient's club or friendly society go to the institution, or should it, as it is the result of a poor man's thrift, be treated with a very light hand and placed on a different footing from any other source of income?*

The proper course for the committee of the hospital to pursue would be to lay down the rule that all patients shall pay according to their means. That is the usual practice at cottage hospitals, and it enables the committee to deal with a case where a man has sick pay by taking from him 2s. 6d. or 5s. a week, as the committee think he can best afford after due enquiry into the circumstances of his family has been made. In this way, should the patient not be able to afford to pay anything, the case can be treated as an object of charity, and in such a case an appeal should be made to the society to which the man belongs asking them to give an annual subscription to the hospital, as is done by most friendly societies.

